# Management of Fish Bone-Induced Liver Abscess with Foreign Body Left In Situ

**DOI:** 10.1155/2019/9075198

**Published:** 2019-06-11

**Authors:** Ryan Burkholder, Hrishikesh Samant

**Affiliations:** ^1^Department of Internal Medicine, Louisiana State University Health Science Center in Shreveport, LA, USA; ^2^Division of Gastroenterology and Hepatology, Louisiana State University Health Science Center in Shreveport, LA, USA

## Abstract

Pyogenic liver abscess, having experienced an evolving pathogenesis over the years, still remains a serious problem with significant morbidity. Iatrogenic and ascending biliary infections are the most common known etiologies for hepatic abscess. Here we report an interesting case of an elderly lady admitted with abdominal pain due to a pyogenic liver abscess in the left liver lobe which was attributed to perforation by an ingested fish bone. The authors also reviewed literature for management for this rare case as there are no standard guidelines. Our patient was successfully treated with antibiotics and percutaneous drainage with foreign body left in situ.

## 1. Introduction

Pyogenic liver abscess is becoming a rare cause of abdominal pain with very low estimated annual incidence at 2.3 cases per 100,000 people [1]. Today, biliary tract disease is still the most common risk factor for development of liver abscess [2]. While accidental ingestion of sharp foreign bodies is common, perforations by foreign body are very rare nowadays due to advances in upper gastrointestinal endoscopic techniques and availability. An etiological diagnosis is often difficult since patients rarely recall ingestion in the absence of odynophagia and can present with nonspecific symptoms including anorexia, vomiting, and weight loss. Here the authors describe a case of a penetration of fish bone from stomach directly into the liver forming a pyogenic liver abscess which successfully responded to drainage and antibiotics. Although various complications of foreign body penetration through the GI tract are reviewed in the literature, few hepatic abscesses due to silent migration of a fish bone have been reported in the Western world.

## 2. Case Report

**Figure 1:**
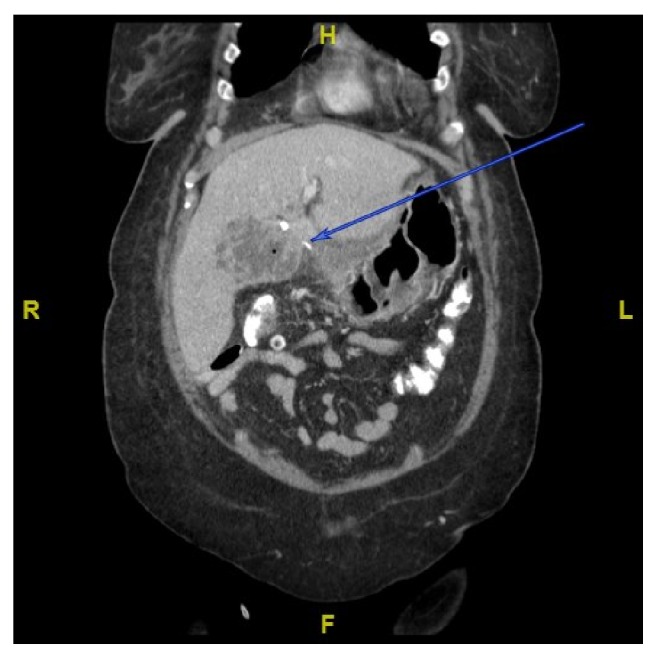
CT on admission illustrating hepatic abscess of left lobe with adjacent foreign body.

**Figure 2:**
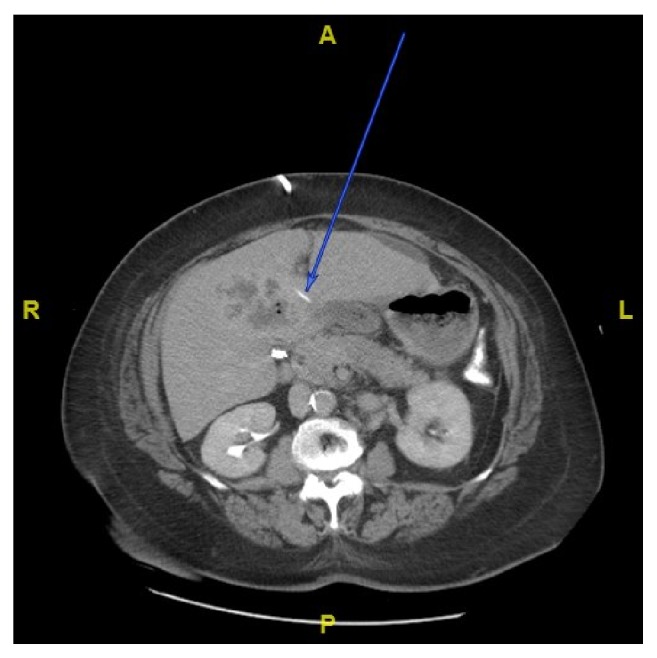
Transverse view indicating foreign body at the level of falciform fissure.

A 64-year-old female with past medical history of hypertension, obesity, and gastroesophageal reflux disease presented to tertiary hospital with complaints of eight days of abdominal pain in the right upper quadrant accompanied by nausea, vomiting, fevers, and diarrhea. On admission, vital signs were notable for BP 130/90, HR 133, RR 18, and temperature of 102.7 F. Physical exam revealed jaundice but did not appreciate abdominal tenderness or guarding. Laboratory investigations showed leukocytosis of 20.2 x 10^9^ cells per liter, total bilirubin 2.4 mg/dL, alkaline phosphatase 114 units per liter (U/L), AST 62 U/L, ALT 59 U/L, and albumin 2.6 g/dL. An abdominal computed tomographic (CT) scan was performed and showed a 6.9 cm heterogeneously enhancing abscess collection within the left hepatic lobe (Figures 1 and 2).

**Figure 3:**
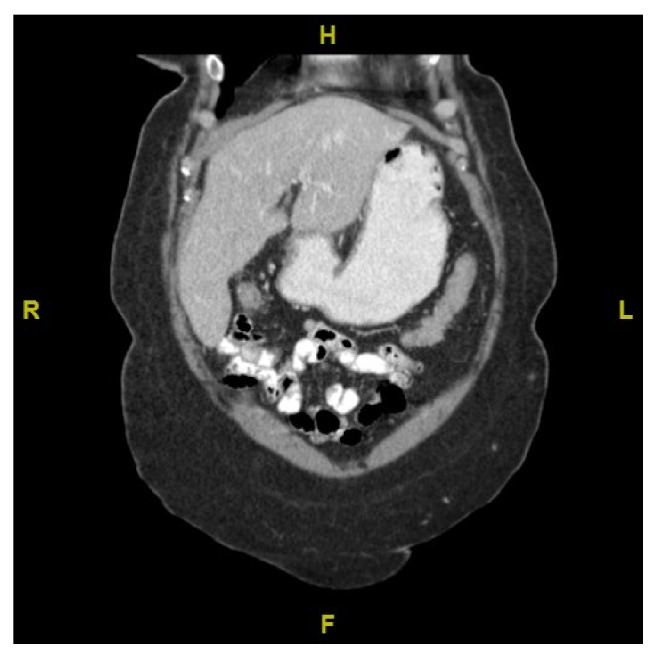
Follow-up CT sagittal view of abdomen at 7 weeks indicates marked improvement.

**Figure 4:**
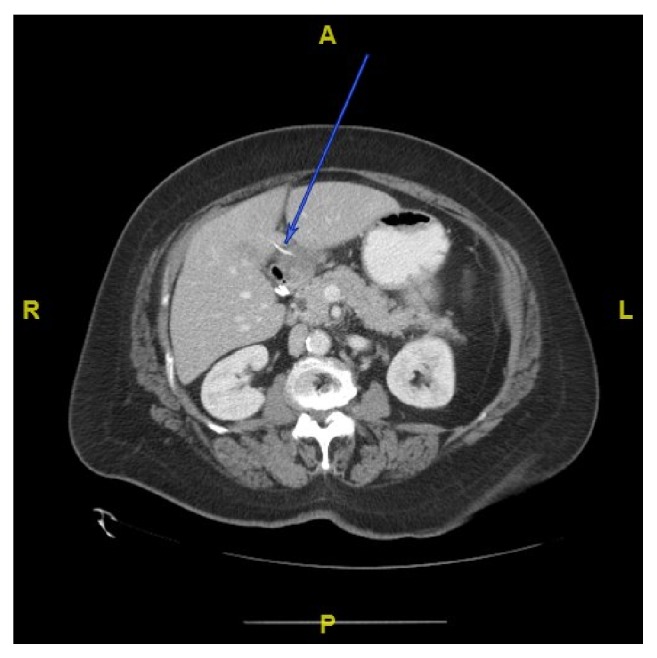
7-week follow-up imaging shows interval improvement of hepatic abscess without any migration of foreign body.

On admission, interventional radiology performed CT-guided percutaneous drainage and placed biliary drain. Abscess cultures returned with alpha hemolytic streptococcus. Due to persistent leukocytosis, a repeat abdominal CT was performed and showed a 2.1 cm fish bone as radiopaque foreign body at the level of the falciform fissure with inflammation tracking to the abscess cavity. Subsequent upper endoscopy failed to visualize a fistulous opening by foreign body in the gastric antrum. The case was discussed in multidisciplinary hepatobiliary conference. Due to rapid clinical improvement with minimal biliary drain output, drains were removed and patient was treated with extended intravenous antibiotics. Patient was seen in clinic 6 weeks later, with no complaints. Repeat CT imaging indicated resolution of hepatic abscess to a size of 1.7x1.3 cm without any further migration of foreign body (Figures 3 and 4).

## 3. Discussion

The first case of hepatic abscess as a result of gastrointestinal perforation caused by a foreign body was published by Lambert in 1898 [3]. The incidence of liver abscesses in developed countries is very low and a thorough workup is warranted to determine potential causes. Pyogenic abscesses can form in the setting of ascending cholangitis, septic emboli via hepatic artery, or following infective suppurative thrombosis of the portal vein.

Sharp objects that account for perforations commonly include toothpicks and dietary foreign bodies including bones, as demonstrated by Goh et al. in a study of 62 patients treated surgically for foreign body perforations [4]. Most sharp foreign bodies are removed endoscopically shortly after ingestion, since they present with odynophagia. Foreign bodies which perforate the gastric wall are quite rare and as suggested by Goh et al., it is possible that a thicker gut wall (stomach and large bowel) causes the foreign body to perforate more gradually. Being in close proximity to the omentum and liver assists in “sealing” the perforation site, therefore no signs of peritonitis or fevers are seen. Since several weeks to months may elapse until such abscesses are recognized, endoscopy may not be helpful.

Hepatic abscess treatment includes antibiotic therapy, percutaneous needle aspiration (PNA), percutaneous drainage (PCD), or surgical drainage. Percutaneous drainage has gained favor recently as an effective method that is cost effective, carrying lower mortality. In a large study by Mangukiya et al., 56% of diagnosed patients with hepatic abscess were treated with aspiration, while abscess of 10 cm or greater underwent percutaneous drainage therapy with excellent results [5]. A recent meta-analysis of 306 patients comparing PNA versus PCD concluded that PCD facilitated higher success rate and reduced the time required to achieve relief and supports a 50% reduction in abscess cavity size [6]. It is interesting to note that several cases have been treated with medical therapy alone and foreign body is left in situ [7, 8]. Our case was unique in that we treated a large 6.9 cm abscess with antibiotics and percutaneous drainage and avoided the need for open surgical intervention.

In conclusion, we believe percutaneous drainage is the appropriate first line treatment in most cases, even in cases of retained of foreign body. Surgical retrieval portends the risk of additional injury to organs, inflammation, and source for sepsis. Our case demonstrates that when the clinical condition of the patient is stable and improving with percutaneous drainage and antibiotics, it may be prudent defer surgical operation.
